# Effects of riociguat in severe experimental pulmonary hypertension

**DOI:** 10.1186/1471-2210-11-S1-P39

**Published:** 2011-08-01

**Authors:** Baktybek Kojonazarov, Michaela Lang, Norbert Weissmann, Friedrich Grimminger, Johannes-Peter Stasch, Werner Seeger, Hossein Ardeschir Ghofrani, Ralph Schermuly

**Affiliations:** 1 University of Giessen Lung Center, Giessen, Germany; 2 Bayer HealthCare, Wuppertal, Germany; 3 Department of Lung Development and Remodeling, Max-Planck Institute for Heart and Lung Research, Bad Nauheim, Germany

## Introduction

The NO-sGC-cGMP signaling pathway is impaired in different cardiovascular diseases, including pulmonary hypertension (PH). Riociguat is the first of a new class of drugs, the soluble guanylate cyclase stimulators. Riociguat has a dual mode of action: it sensitizes sGC to the body’s own NO and can also increase sGC activity in the absence of NO, causing vasorelaxation, anti-proliferation and anti-fibrotic effects.

The aim of the study was to investigate the effects of riociguat as compared to the PDE5 inhibitor sildenafil on pulmonary vascular remodeling in severe experimental PH.

## Methods

Angioproliferative PH was induced in rats by combined exposure to the vascular endothelial growth factor-receptor antagonist SU5416 and hypoxia at 10%O2 (SU+HOX). Twenty-one days thereafter, rats were randomized for treatment with riociguat (10 mg/kg), sildenafil (50 mg/kg) or vehicle for the next 14 days. Echocardiography and invasive hemodynamic measurements were performed. Pulmonary vascular remodeling was assessed by histomorphometric analysis.

## Results

In rats with established PH, right ventricular systolic pressure (RVSP) was significantly decreased by treatment with riociguat to 73±4 mmHg (p<0.01) and sildenafil to 80±3 mmHg (p<0.05) as compared to placebo (89±3 mmHg). No significant difference in systemic arterial pressure was detected between placebo and treated animals. Both compounds significantly decreased RV hypertrophy and improved RV function by normalization of TAPSE and myocardial performance index, but effects of riociguat were more pronounced. Riociguat significantly reduced the proportion of occluded arteries and increased proportion of opened arteries and decreased neointima/media ratio.

## Conclusion

We demonstrated that riociguat effectively suppresses pulmonary vascular remodeling and significantly improves RV function in an experimental model of severe PH.

